# A Novel Method of Characterizing Genetic Sequences: Genome Space with Biological Distance and Applications

**DOI:** 10.1371/journal.pone.0017293

**Published:** 2011-03-02

**Authors:** Mo Deng, Chenglong Yu, Qian Liang, Rong L. He, Stephen S.-T. Yau

**Affiliations:** 1 Department of Mathematics, Statistics and Computer Science, University of Illinois at Chicago, Chicago, Illinois, United States of America; 2 The Institute of Mathematical Sciences, The Chinese University of Hong Kong, Shatin, Hong Kong, People's Republic of China; 3 Department of Biological Sciences, Chicago State University, Chicago, Illinois, United States of America; Midwestern University, United States of America

## Abstract

**Background:**

Most existing methods for phylogenetic analysis involve developing an evolutionary model and then using some type of computational algorithm to perform multiple sequence alignment. There are two problems with this approach: (1) different evolutionary models can lead to different results, and (2) the computation time required for multiple alignments makes it impossible to analyse the phylogeny of a whole genome. This motivates us to create a new approach to characterize genetic sequences.

**Methodology:**

To each DNA sequence, we associate a natural vector based on the distributions of nucleotides. This produces a one-to-one correspondence between the DNA sequence and its natural vector. We define the distance between two DNA sequences to be the distance between their associated natural vectors. This creates a genome space with a biological distance which makes global comparison of genomes with same topology possible. We use our proposed method to analyze the genomes of the new influenza A (H1N1) virus, human rhinoviruses (HRV) and mammalian mitochondrial. The result shows that a triple-reassortant swine virus circulating in North America and the Eurasian swine virus belong to the lineage of the influenza A (H1N1) virus. For the HRV and mammalian mitochondrial genomes, the results coincide with biologists' analyses.

**Conclusions:**

Our approach provides a powerful new tool for analyzing and annotating genomes and their phylogenetic relationships. Whole or partial genomes can be handled more easily and more quickly than using multiple alignment methods. Once a genome space has been constructed, it can be stored in a database. There is no need to reconstruct the genome space for subsequent applications, whereas in multiple alignment methods, realignment is needed to add new sequences. Furthermore, one can make a global comparison of all genomes simultaneously, which no other existing method can achieve.

## Introduction

Computational and statistical methods to cluster the DNA or protein sequences have been successfully applied in clustering DNA, protein sequences and microarray data [1]–[8]. Yau and his group showed that the genomic space method was an efficient way to cluster the DNA or protein sequences [9]–[13]. In [9], [11] each nucleic base or amino acid was assigned a specific value. For example, nucleic base adenine A was assigned to the pair 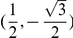 [9]. This method can be used successfully to represent a DNA sequence in the Cartesian coordinate plane, however the nucleotides are artificially assigned to specific values which are not inherently related to DNA or protein sequences. In contrast, the parameters used in this work are natural because they are based on the numbers and distributions of nucleotides in the sequence.

In this paper we propose a method of characterizing DNA sequences, which uses a specific mathematical description of distributions of nucleotides in a DNA sequence that represents the biological information in the sequence. To each DNA sequence we associate a natural sequence of parameters, called a natural vector, describing the numbers and distributions of nucleotides in the sequence. We show that the correspondence between a natural vector and a DNA sequence is one-to-one. A natural distance between two genes is the distance between their corresponding natural vectors. This creates a genome space with biological distance, which allows us to do phylogenetic analysis in the most natural and easy manner. This alignment-free method is much faster than conventional multiple sequence alignment methods. Multiple sequence alignment (MSA) can be seen as a generalization of pair-wise sequence alignment, in which, instead of aligning two sequences, k sequences are aligned simultaneously. MSA is the most powerful method to analyze the genetic sequences and most of state-of-art algorithms are constructed based on it [14]–[16]. It is however, an NP-hard computational optimization problem which is implausible for a huge amount of sequences [17].

**Figure 1:**
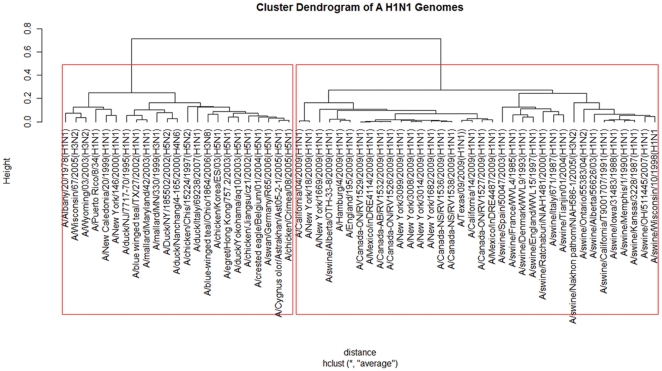
Genome analysis. We apply our method to analyze 59 influenza viruses based on their whole genomes. The natural vector and the hierarchical clustering methods are used to reconstruct the phylogenetic tree for nucleotide sequences of the whole genome sequences of selected influenza viruses. The selected viruses are chosen to be representative from among all available relevant sequences in GenBank. Sequences have both high and low divergence to avoid biasing the distribution of branch lengths. Strains are representative of the major gene lineages from different hosts. The robustness of individual nodes of the tree is assessed using a bootstrap resampling analysis with 1000 replicates shown in Supporting Information S1. From this figure, we can clearly see that new influenza A (H1N1) viruses originate from North American triple-reassortant swine virus and Eurasian classical swine virus lineage. We note that (A/swine/Nakhon pathom/NIAH586-1/2005(H3N2)), (A/duck/Nanchang/4-165/2000(H4N6)) and American avian (A/blue-winged teal/Ohio/1864/2006(H3N8)) are not clustered with A (H1N1) genomes from the same geographical regions respectively. This result is caused by the different structures of these genomes and the traditional A (H1N1) subtypes. In addition, we check the distance matrix of these genomes obtained by natural vectors and the result shows that (A/duck/Nanchang/4-165/2000(H4N6)) is the closest to A/duck/NY/185502/2002(H5N2). Meanwhile, A/blue-winged teal/Ohio/1864/2006(H3N8) is the closest to A/chicken/Korea/ES/03(H5N1) and A/egret/Hong Kong/757.2/2003(H5N1) respectively, which means that A/blue-winged teal/Ohio/1864/2006(H3N8) is evolutionary related with H5N1 avian virus outbreak in Asian countries from 2003 to 2006. As for A/swine/Nakhon pathom/NIAH586-1/2005(H3N2), it is the closest to A/swine/Tianjin/01/2004(H1N1) and then to A/swine/Ontario/55383/04(H1N2) with and A/swine/OH/511445/2007(H1N1). This H3N2 is the closest related to Eurasian swine even if it is clustered within American swine clade (The large distance matrix data is not shown and available upon request).

**Figure 2:**
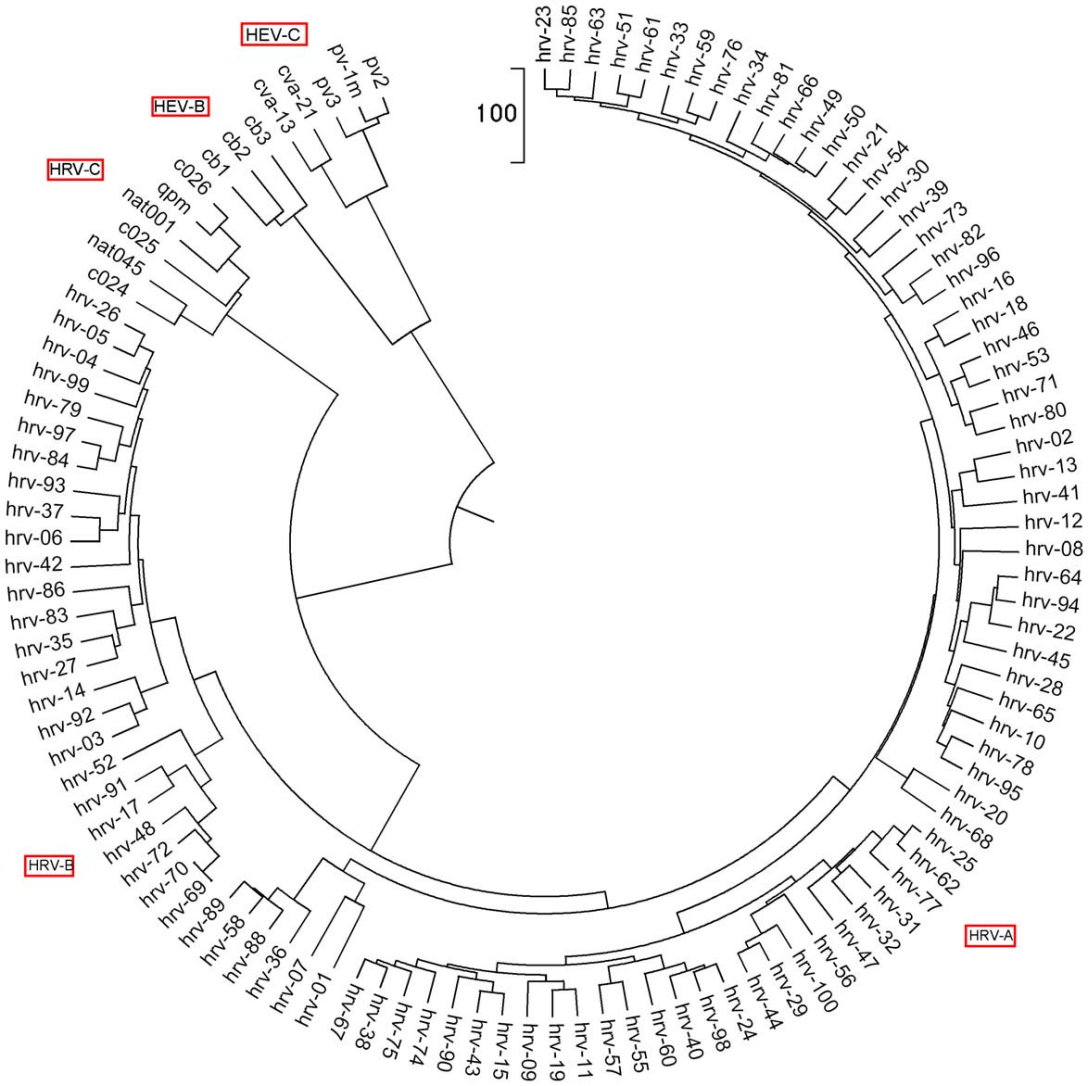
The Natural vector method is used for clustering the HRV genome virus at the whole genome level. All HRV data are provided in [19] and the corresponding details are described in Supporting Information S1. This figure shows relationships between all known HRV serotypes created on the basis of full genome sequences. The HEV-B, C sequences are used as outgroups. The five clusters listed around the circular tree, HRV-C, HRV-B, HRV-A, HEV-B and HEV-C are separated clearly (HEV-B, C are outgroups) by using MEGA software [27]. This clustering result is the same as Palmenberg et al's result shown in figure S6a in their paper [19]. This method only needs 18 seconds to obtain this clustering result while it takes more than 19 hours for the multiple alignment method on the same dataset.

**Figure 3:**
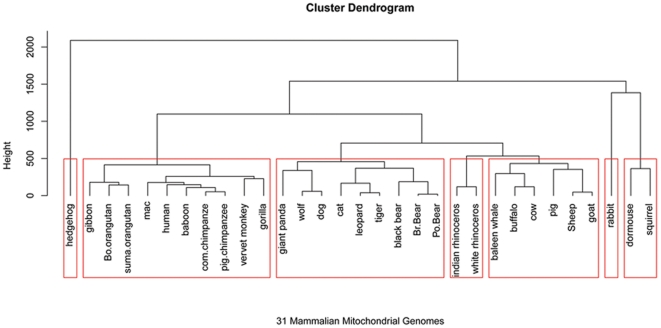
Genome analysis on 31 mammalian mitochondrial genomes. We applied our method to analyze 31 mammalian mitochondrial genomes. From our clustering analysis, we can see that all 31 genomes are correctly clustered into 7 known clusters: Erinaceomorpha (cluster 1), Primates (cluster 2), Carnivore (cluster 3), Perissodactyla (cluster 4), Cetacea and Artiodactyla (cluster 5), Lagomorpha (cluster 6), Rodentia (cluster 7). Data are provided in Table 1. For the primates and carnivores subgroups, the clades are a little different from those obtained by using mitochondrial DNA coding sequences. In this experiment, we use the whole genome sequences containing all tRNA, sRNA, polypeptide-encoding genes and D-loop rather than mtDNA coding sequences, which may lead slightly different results. In fact, the distance matrix obtained by natural vectors shows that human is the closest to c.chimpanzee and p.chimpanzee with the distance of 994123.7 and 1346597.8 respectively, while giant panda is the closest to black bear with the distance of 2468063, although they are not clustered together.

**Figure 4:**
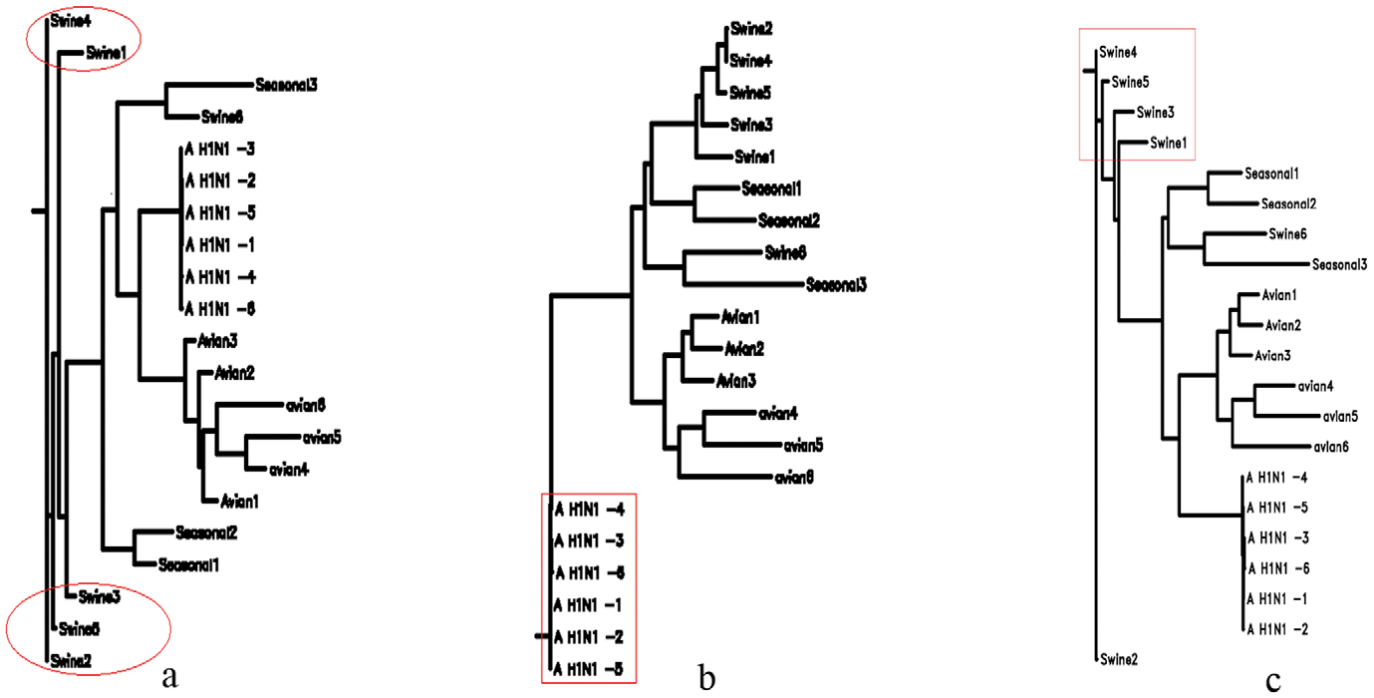
Phylogenetic trees are reconstructed by using the maximum likelihood (ML) alignment method with Jukes-Cantor model, the neighbor-joining (NJ) method with the Kimura 2 parameter model and with the Jukes-Cantor model. It is clear that swine flu viruses are not clustered correctly using the ML method (figure 4(a)). The NJ method with the Kimura and Jukes-Cantor models yields totally different phylogenetic trees. The Kimura model fails to distinguish the origin of A H1N1 virus since A H1N1 genomes are all very far away from other genomes (figure 4(b)), while the Jukes-Cantor model fails to cluster swine flu viruses correctly(figure 4(c)). The data is described in Supporting Information S1.

For our first application, we analysed the new influenza A (H1N1) virus based on the whole genome (figure 1). The previous research of A (H1N1) only focuses on individual segmented genes [18]. We analyze the whole genome and individual genes as well. The influenza A virus (H1N1) genome contains 8 genes: polymerase PB2, PB1, PA, hemagglutinin HA, neuraminidase NA, nucleocapsid NP, matrix protein MP and nonstructural gene NS. The results of our whole genome, natural vector method analysis show that the lineage of the influenza A (H1N1) virus includes a triple-reassortant swine virus circulating in North America and the Eurasian swine virus, but does not include either human seasonal influenza and avian viruses. In addition, our results of analysis on each individual gene coincide with Garten et al [18]. We also analyzed the whole genomes of human rhinoviruses (HRV), which cause serious upper and lower respiratory tract disease worldwide. The result, shown in the phylogenetic tree constructed from known HRV whole genomes, demonstrates that the five clusters HRV-A, HRV-B, HRV-C, HEV-B and HEV-C are clearly separated from each other (figure 2). This result coincides with Palmenberg et al. 's result [19]. As another biological application, a dataset of 31 mammalian mitochondrial genomes was analyzed by our method (figure 3). Our approach also considers circular genomes. The result shows that these 31 genomes are well clustered. The natural vector method gives us the natural distance between two genes or genomes while the distance obtained from other methods depends on the choice of evolutionary models. As a result, there are big variations among the phylogenetic trees obtained from various models. Here we performed the maximum likelihood (ML) method and neighbor-joining (NJ) method on a dataset of 21 flu virus genomes. We found that the swine flu viruses are not clustered correctly by using the ML method with the J-C model (figure 4(a)) and the origin of A H1N1 virus is not clear by NJ method with Kimura model (figure 4(b)) since A H1N1 genomes are all very far away from other genomes. The result obtained by the NJ method with the Jukes-Cantor model (figure 4(c)) fails to cluster swine flu virus correctly and the result is totally different from that by the Kimura model (figure 4(b)).

**Table 1:** Description of 31 mammalian mitochondrial genome data in figure 3.

Number	Genome name on the tree	GenBank ID
1	Human	V00662
2	pigmy chimpanzee	D38116
3	common chimpanzee	D38113
4	Gibbon	X99256
5	Baboon	Y18001
6	vervet monkey	AY863426
7	Macaca thibetana	NC 002764
8	bornean orang-utan	D38115
9	sumatran orang-utan	NC 002083
10	Gorilla	D38114
11	Cat	U20753
12	Dog	U96639
13	Pig	AJ002189
14	Sheep	AF010406
15	Goat	AF533441
16	Cow	V00654
17	Buffalo	AY488491
18	Wolf	EU442884
19	Tiger	EF551003
20	Leopard	EF551002
21	indian rhinoceros	X97336
22	white rhinoceros	Y07726
23	black bear	DQ402478
24	brown bear	AF303110
25	polar bear	AF303111
26	giant panda	EF212882
27	Rabbit	AJ001588
28	Hedgehog	X88898
29	Dormouse	AJ001562
30	Squirrel	AJ238588
31	blue whale	X72204

## Results

As an application, we first use our method to analyze the new influenza A (H1N1) virus. Recent reports of widespread transmission of swine-origin influenza A (H1N1) viruses in humans in Mexico, the United States, and elsewhere, highlighted this ever-present threat to global public health [20]. Much effort has been made by using the experimental method and many important results have been obtained in the past [18], [21]. Pigs have been hypothesized to act as a mixing vessel for the reassortment of avian, swine, and human influenza viruses and might play an important role in the emergence of novel influenza viruses capable of causing a human pandemic [22]–[24]. There were many reports of recent transmissions of swine influenza viruses in humans [25]. The new strain was initially described as triple reassortants of viruses from pigs, humans, and birds, called triple-reassortant swine influenza A (H1) viruses, which have circulated in pigs for more than a decade [20]. Subsequent analysis suggested it was a reassortment of just two strains, both found in swine [21]. Although initial reports identified the new strain as swine influenza (i.e., a zoonosis originating in swine), its origin is unknown from the point of view of whole genomes. Here we used our proposed method to verify the origin of A (H1N1) genomes. To demonstrate that our natural vector can be truly useful for answering biological questions, we performed hierarchical clustering analysis on the natural vectors of the genes of the swine influenza A (H1N1) virus. The Euclidean distance was used to measure the distance between natural vectors. Genomes of the outbreak of swine influenza A (H1N1), North American and Eurasian swine influenza virus genomes, avian and human seasonal influenza virus genomes were analyzed. Each complete genome contains 8 complete gene-coding segments. So we used a 96-dimensional natural vector to represent a whole genome since each segment can be characterized very well by using a 12-dimensional natural vector. Based on our novel mathematical method and result, we can predict that the genome of new swine influenza A (H1N1) is similar to swine viruses rather than human seasonal influenza and avian viruses. Using the natural vector method and clustering method, we have reconstructed the complex reassortment history of the outbreak of swine influenza A (H1N1), summarized in figure 1. Our analysis showed that the swine influenza A (H1N1) genome was nested within a well-established triple-reassortant swine influenza A and Eurasian swine influenza A lineage (that is, a lineage circulating primarily in swine before the current outbreak). In addition, we also analyzed 8 segments: polymerase PB2, PB1, PA, hemagglutinin HA, neuraminidase NA, nucleocapsid NP, matrix protein MP and nonstructural gene NS respectively in A H1N1 genome. Our results showed that HA, NP, NS genes resemble those of classical swine influenza A viruses and PB2, PB1, PA genes resemble those of triple-reassortant swine influenza A viruses circulating in pigs in North America while the genes NA and MP are most closely related to those in influenza A viruses circulating in swine populations in Eurasia. The clustering results of these 8 gene segments obtained by our method coincides with the phylogenetic analysis results from Garten et al. [18] and Novel Swine-Origin Influenza A (H1N1) Virus Investigation Team [21]. These conclusions have been widely accepted by other scientists [35] in the scientific community. Therefore, this result shows that Kou et al.'s conclusion [26] was not fully convincing since they concluded that PB2 and PA genes came from avian influenza virus and PB1 from human seasonal influenza virus. As an illustration, the phylogenetic analysis result of PB2 is shown in Supporting Information S1. The rest results of seven individual segments are available from the author upon request. In this biological experiment, 12 dimensional natural vectors, <
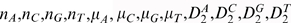
> were used for clustering the swine influenza A (H1N1) based on the gene sequences, since the higher moments in the natural vector were too small to play a role when *n* is large. The gene and genome data are provided in the section of Supporting Information. They can be downloaded from Flu Database of GenBank (http://www.ncbi.nlm.nih.gov/genomes/FLU/FLU.html).

In addition, we applied our approach to study another group of viruses, human rhinovirus (HRV). Infection by HRV is a major cause of upper and lower respiratory disease worldwide and displays considerable phenotypic variation. Recently, Palmenberg et al. [19] reported a comprehensive sequencing and analyzed result for all known HRV genomes based on the whole genome. In their article, the authors used the multiple alignment method to reconstruct the evolutionary tree. In that tree, five groups HRV-A, HRV-B, HRV-C, HEV-B and HEV-C were clearly identified. We used our natural vector method to perform clustering analysis for the same dataset (Supporting Information S1). We associated each whole genome sequence with a natural vector, and then by computing the Euclidean distances among these natural vectors we obtained the evolutionary tree (figure 2) for all HRVs. MEGA software was used to draw the tree [27]. According to our result, the five clusters HRV-A, HRV-B, HRV-C, HEV-B, and HEV-C are clearly separated from each other. Our method takes only 18 seconds to complete the clustering analysis result while it takes more than 19 hours for the multiple alignment method. Both methods yield the same clustering result.

As another biological application, we consider the phylogeny of mitochondrial genomes. Mitochondrial DNA is not highly conserved and has a rapid mutation rate, thus it is very useful for studying the evolutionary relationships of organisms [28]. We extracted 31 representative cases of complete mammalian mitochondrial genome sequences from the GenBank, each of which has length of more than 16000 nucleotides. Moreover, they have double-strand and circular structures. As mentioned in the section of Materials and Methods, we just treat them as the single-strand (by using the heavy strand) circular genomes, because the gene contest of both strands of these genomes is already known. For this case, we treat every point as the starting point in this circular sequence of length n, and then we get n linear single-strand genomes. For every linear single-strand genome sequence, we can compute its (*n+4*)-dimensional natural vector. Then we take the average to get a normalized vector <
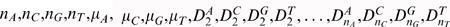
>. Here we use the first 16 moments of the natural vector, i.e., <
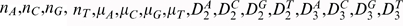
> to characterize these 31 genomes. By computing the Euclidean distances between these points, we obtain the distance matrix for these 31 organisms. The phylogenetic tree is shown in figure 3. The result shows these 31 genomes are well clustered into 7 clusters: Erinaceomorpha, Primates, Carnivore, Perissodactyla, Cetacea and Artiodactyla, Lagomorpha and Rodentia, where Cetacea and Artiodactyla form a sister-group since they are grouped together. This result coincides with the conclusion found by Liu et al [29], Raina et al [30], and Kullberg et al [31].

In the Supporting Information S1, we discuss the distribution of distance of the corresponding natural vectors under controlled simulation. 1000 simulated sequences are generated by shuffling a real genome sequence and the distribution of pair-wise distance of the natural vectors Ls is plotted. We also consider the following simulated experiment on gene rearrangement (please refer to Supporting Information S1 for more details). We choose a human mitochondrial genome denoted by human (GenBank ID: V00662), and then invert its two genes ATPase 6 and Cytochrome oxidase to get a simulated genome, which is denoted by human-inv. We can treat this new simulated genome as the result of the inversion of genes from the original human mitochondrial genome. Next we randomly generate a genome sequence which has the same length and nucleotide content as the original human genome, which we denote by human-ran. Thus, we have 3 genomes of the same length and nucleotide content: human, human-inv and human-ran. In addition, we also choose another mitochondrial genome, chimpanzee as comparison since chimpanzee and human are so close evolutionarily. By using the 16-dimmensional natural vector, we calculate the distance among these 4 genomes and get a distance matrix in the Supporting Information S1. We find that the distance between human and human-inv is as small as 1.7 units. This means that the gene-inverted genome still has a very short distance to the original genome even if some gene rearrangement happens in this genome. As a result, the original genome and its gene rearrangement genome cannot be treated separately by using our method since the evolutionarily very close genome to human is chimpanzee which has the distance of 16.88 units to human. More importantly, this simulation demonstrates that our method can be applied to do clustering or phylogenetic analysis. If two genetic sequences are close in the distance, they should be close in the evolutionary tree. For example, the distance between two real genomes, human and chimpanzee is 16.88 units. Since the distance between the original human genome and all shuffled genomes ranges from 114.49 to 1009.00 with the mean value of 190.60 units (Supporting Information S1), all those randomly shuffled sequences cannot be clustered together with the human. Here we use standard multiple alignments with substitutions and indels on 21 flu virus genomes (figure 4). We performed the multiple alignment method using ClustalX and Phylip to draw the phylogenetic tree for the dataset consisting of 6 influenza A (H1N1) genomes, 6 swine flu virus genomes, 6 avian virus genomes and 3 human seasonal flu virus genomes (Please see Supporting Information S1 for the description of these genomes). These genomes are selected from the dataset shown in figure 1. We reconstructed the phylogenetic trees by maximum likelihood method and neighbor-joining method with different models shown in figure 4(a, b, c). The phylogenetic result obtained by using maximum likelihood method (figure 4(a)) showed that the swine flu virus genomes were not clustered correctly. The result by using neighbor-joining method with Kimura model did not obviously show the origin of A (H1N1) genomes (figure 4(b)), because all A (H1N1) genomes were very far away from other genomes. Besides, the result obtained from neighbour-joining method with Jukes-Cantor model also fails to cluster swine flu viruses correctly. Our method showed that influenza A (H1N1) genomes are close to swine flu virus genomes (figure 1). Therefore, we predict that A (H1N1) genomes are originally from swine flu virus genome lineage (triple-reassortant North American and Eurasian swine virus genomes). The advantage of our method is the quick speed. It took us 60 minutes 31 seconds to finish the complete alignment for these 21 sequences by using multiple alignment method [14] whereas we just need 6 seconds to get the result.

In order to compare the computation time of the natural vector method and state-of-the-art methods ClustalW2, MUSCLE and MAFFT [14], [15], [16], we performed the test on two sets of sequences. The first set included 8 datasets. The datasets contain 10, 20, 30, 40, 50, 60, 70 and 80 sequences respectively, where the lengths of all the sequences are around 4000. Another set was created with 8 datasets. Each of these dataset had 40 sequences. The lengths of all sequences in these 8 datasets were 1000, 2000, 3000, 4000, 5000, 6000, 7000 and 8000 respectively. We built the trees on each of the datasets by using the four methods and recorded the time that each method took. The results shown in Supporting Information S1 demonstrate that natural vector method is much faster than the other three methods. The time of our method increases linearly as the number of sequences or the length of sequences increase, whereas the time for other three methods increases much faster. The actual time differences are much larger than the visual differences in the figure since we are using the logarithm of time as the label of y-axis.

## Discussion

In this paper, we report a new mathematical method to characterize a genetic sequence as a natural vector so we can perform clustering analysis and create a phylogenetic tree based on it. A natural vector system to represent a DNA sequence is introduced, and the correspondence between a DNA sequence and its natural vector is mathematically proved to be one-to-one. With this natural vector system, each genome sequence can be represented as a multidimensional vector. Genomes with a close evolutionary relationship and similar properties are plotted close to each other when we construct the phylogenetic tree. Thus, it will provide a new powerful tool for analyzing and annotating genomes and their phylogenetic relationships. Our method is easier and quicker in handling whole or partial genomes than multiple alignment methods. There are four major advantages to our method: (1) once a genome space has been constructed, it can be stored in a database. There is no need to reconstruct the genome space for any subsequent application, whereas in multiple alignment methods, realignment is needed for adding new sequences. (2) One can perform global comparison of all genomes simultaneously, which no other existing method can achieve. (3) Our method is quicker than alignment methods and easier to manipulate, because not all dimensions of natural vectors are needed for computing. Instead, the first several dimensions of natural vectors are good enough to cluster DNA sequences or genomes. Generally, we select the first *N* dimensions such that the clustering result remains stable even if we choose higher moments. N = 12 in our experiments is good enough to characterise all sequences. We can compare all genes, DNA and genome sequences with different lengths by truncating all different (n+4) natural vectors into the same number of dimensions. The one-to-one correspondence between the truncated natural vectors (with 12 or more dimensions) and sequences is still valid. (4) The current standard methods involve the evolutionary models. The different choices of these evolutionary models can lead to inconsistent results (figure 4 (a, b, c)). There is no evidence to show which model can best fit all biological datasets without human intervention (likelihood ratio test used as a priority). This motivates us to create a new mathematical method without any model. Our method does not involve these models and it totally depends on the natural vectors constructed from the whole sequences. Therefore, this method is stable, natural and produces a unique clustering or phylogenetic result.

Although the natural vector method can be used to reconstruct the phylogenetic trees of DNA sequences, genes and whole genomes, this method may not be a suitab le substitute for local multiple sequence alignment when one wants to identify the similarity of genomic subsequences and does not know a priori which subsequences to identify.

## Materials and Methods

### Natural vector of a DNA sequence

Let us first introduce the definition of normalized central moments which is the most important part of natural vector method. Normalized central moments are defined as follows:
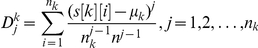
where *k* = A, C, G, T. Here, 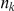 denotes the number of nucleotide k in the DNA sequence and n is the length of the DNA sequence. 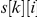 is the distance from the first nucleotide (regarded as origin) to the ith nucleotide k in the DNA sequence. 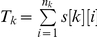 denotes the total distance of each set of A, C, G, T from the origin, k = A, C, G, T. 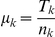, which is the mean value of the distances of the nucleic bases from the origin. Therefore, we have the sequence of central moments: <
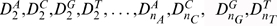
>, Observe that these are natural parameters associated to a DNA sequence.

Our method described below is to give a complete understanding of the distribution of four nucleotides A, C, G and T.

The quantities of the four nucleotides: A, C, G and T of a DNA sequence are chosen as the first four parameters of the natural vector. Four integers 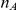, 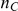, 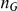 and 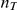 denote the numbers of nucleic bases A, C, G and T in the DNA sequence.

The second group of numerical parameters which are a part of the natural vector are the mean values of total distance, one for each of the four nucleotide bases: 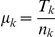, *k = A, C, G and T*.

As a simple illustration for the DNA sequence GTTCAATACT: The total distance of A is 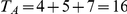, since the distance of origin to the three nucleotide As is 4, 5 and 7 respectively. Then 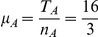. The arithmetic mean value of total distance for other nucleotide base G, C and T can be obtained in the same way.

The final group of parameters that we include in the natural vector are composed of normalized central moments. The first normalized central moment is:
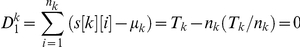
Because the first central moment is zero, we start with the second normalized central moment. The second normalized central moment is the variance of the distance distribution for each base: 
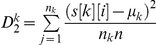
, where *k* = A, C, G, T. If the distribution of each nucleotide base is different, DNA sequences cannot be same even though they may have the same nucleotide contents and the same total distance measurement. Therefore, the information about distribution has also been included in the natural vector. As described above, each subset of numerical parameters is not sufficient to annotate DNA sequences. However, the combined numerical parameters are sufficient to characterize each DNA sequence. So the natural vector is given as follows:
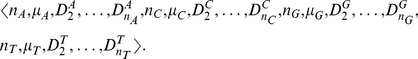
In order to express the vector elegantly and prove the theorem easily, we rewrite it as follows:
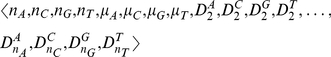
(1)Alternatively, the natural vector can be written as
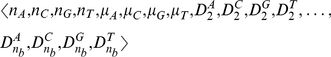
(2)where 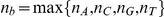. By the definition, 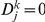, if 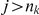. For instance, this case happens when we compute <
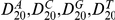
> if there are 19 A, 15 C, 21 G and 22 T in the sequence. Therefore, the 20^th^ moment will be <0, 0, 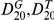
>. The natural vector is obtained by concatenating the first group of parameters (the number of each base) and the second group of parameters (the mean value of total distance of each base) to the normalized central moments.

Obviously, higher moments converge to 0 for a random generated sequence since for any given *k*,
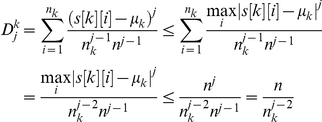
It is clear that 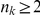, otherwise, 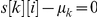. From the viewpoint of probability, suppose that the expectation value of any nucleic base is 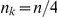 (uniform distribution) for a sequence with given length *n*, therefore
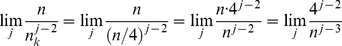
Clearly, this limit goes to 0 as *j* approaches 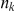. In molecular biology, we can simply discuss the number 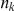 from GC-content. GC-content (or guanine-cytosine content), in molecular biology, is the percentage of nitrogenous bases on a DNA molecule which are either guanine or cytosine. GC content is found to be variable with different organisms. Because of the nature of the genetic code, it is however virtually impossible for an organism to have a genome with a GC-content approaching either 0% or 100%. A species with an extremely low GC-content is Plasmodium falciparum (GC% = ∼20%) [32]. Therefore, for any simulated dataset or biological dataset, 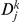 always converges to 0 when *j* approaches 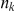. For a simulation, we generate a random sequence with length of 10000 nucleotides by using Hidden Markov model (Matlab, bioinformatic toolbox). The simulated sequence contains 2345 A, 2761 C, 2544 G and 2350 T. For adenines, 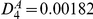, i.e., higher normalized central moments starting from 4^th^ moment will converge to 0.

We have used natural vector to obtain a good numerical characterization of DNA sequence. We now discuss the construction of natural vectors of genomes. Generally, for a linear single-strand genome, we treat it as a linear DNA sequence while we treat every point as the starting point and then take average for circular single-strand genomes. For general double-strand genomes, we treat them as two single-strand genomes and then take average. More details are discussed in Supporting Information S1.

### Theorem

One of the most important things in this paper is that we can prove that the correspondence between a DNA sequence and its natural vector is one-to-one (see Supporting Information S1).

**Theorem:** Suppose a DNA sequence has *n* nucleotides. Then the correspondence between a DNA sequence and its natural vector <
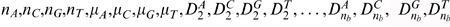
> is one-to-one, where n = 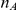
+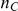
+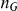
+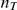.

The Euclidean distance between two sequences 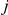 and 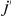 is defined as the distance of their corresponding natural vectors: 
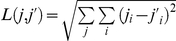
, where *i* = A, C, G, T; *j* = *n*,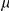,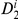,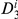,…,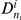. 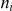 is the number of each A, C, G, T.

We used Matlab to calculate the natural vectors of genes and genomes. The package HCLUST of R language (for algorithmic details, please refer to [33]) was used to perform the hierarchical cluster analysis of genomes and genes. R package PVCLUST [34] was used to calculate approximately unbiased p-value and bootstrap probability value by multiscale bootstrap resampling and draw the standard error plot. The codes are available from the author upon request.

## Supporting Information

Supporting Information S1 Supporting information S1 contains the complete proof of the correspondence theorem, the bootstrapping analysis on A H1N1 genomes, distribution of the distance between each pair of random shuffled genomes under simulation, clustering of the segmented gene PB2, computational time chart of natural vector method, ClustalW2, MUSCLE and MAFFT, and the Genbank ID of the data used in this paper.
(PDF)

## References

[1] Amano K, Nakamura H (2003). Self-organizing clustering: a novel non-hierarchical method for clustering large amount of DNA sequences.. Genome Inform.

[2] Emrich SJ, Kalyanaraman A, Aluru S, Aluru S (2006). Algorithms for large-scale clustering and assembly of biological sequence data.. Handbook of Computational Molecular Biology.

[3] FitzGerald PC, Shlyakhtenko A, Mir A, Vinson C (2004). Clustering of DNA sequences in human promoters.. Genome Res.

[4] Waterman SM (1995). Introduction to computational biology: maps, sequences and genomes.

[5] Abe T, Kanaya S, Kinouchi M, Ichiba Y, Kozuki T (2003). Informatics for unveiling hidden genome signatures.. Genome Research.

[6] Chuzhanova NA, Jones AJ, Margetts S (1998). Feature selection for genetic sequence classification.. Bioinformatics.

[7] Karlin S, Ladunga I (1994). Comparisons of eukaryotic genomic sequences.. Proc Natl Acad Sci U S A.

[8] Nakashima H, Ota M, Nishikawa K, Ooi T (1998). Genes from nine genomes are separated into their organisms in the dinucleotide composition space.. DNA Res.

[9] Yau S, Wang J, Niknejad A, Lu C, Jin N (2003). DNA sequence representation without degeneracy.. Nucl Acids Res.

[10] Liu L, Ho Y-K, Yau S (2006). Clustering DNA sequences by feature vectors.. Mol Phyl Evol.

[11] Yau SS-T, Yu C, He R (2008). A protein map and its application.. DNA and Cell Biol.

[12] Carr K, Murray E, Armah E, He RL, Yau SS-T (2010). A rapid method for characterization of protein relatedness using feature vectors.. PLoS One.

[13] Yu C, Liang Q, Yin C, He RL, Yau SS-T (2010). A novel construction of genome space with biological geometry.. DNA Res.

[14] Larkin MA, Blackshields G, Brown NP, Chenna R, McGettigan PA (2007). Clustal W and Clustal X version 2.0.. Bioinformatics.

[15] Edgar RC (2004). MUSCLE: a multiple sequence alignment method with reduced time and space complexity.. BMC Bioinformatics.

[16] Katoh K, Misawa K, Kuma K, Miyata T (2002). MAFFT: a novel method for rapid multiple sequence alignment based on fast Fourier transform.. Nucl Acids Res.

[17] Wang L, Jiang T (1994). On the complexity of multiple sequence alignment.. J Comput Biol.

[18] Garten RJ, Davis CT, Russell CA, Shu B, Lindstrom S (2009). Antigenic and Genetic Characteristics of Swine-Origin 2009 A (H1N1) Influenza Viruses Circulating in Humans.. Science.

[19] Palmenberg A, Spiro D, Kuzmickas R, Wang S, Djikeng A (2009). Sequencing and analyses of all known human rhinovirus genomes reveal structure and evolution.. Science.

[20] Shinde V, Bridges CB, Uyeki TM, Shu B, Balish A (2009). Triple-reassortant swine influenza A (H1) in humans in the United States, 2005–2009.. New Engl J Med.

[21] Novel Swine-Origin Influenza A (H1N1) Virus Investigation Team (2009). Emergence of a novel swine-origin influenza A (H1N1) virus in humans.. New Engl J Med.

[22] Scholtissek C (1990). Pigs as ‘mixing vessels’ for the creation of new pandemic influenza A viruses.. Med Princ Pract.

[23] Ito T, Couceiro JN, Kelm S, Baum LG, Krauss S (1998). Molecular basis for the generation in pigs of influenza A viruses with pandemic potential.. J Virol.

[24] Ma W, Kahn RE, Richt JA (2009). The pig as a mixing vessel for influenza viruses: human and veterinary implications.. J Mol Genet Med.

[25] Belshe RB (2009). Implications of the emergence of a novel H1 Influenza virus.. New Engl J Med.

[26] Kou Z, Hu S, Li T (2009). Genome evolution of novel influenza A (H1N1) viruses in humans.. Chin Sci Bul.

[27] Kumar S, Dudley J, Nei M, Tamura K (2008). MEGA: A biologist-centric software for evolutionary analysis of DNA and protein sequences.. Brief Bioinform.

[28] Brown WM, Prager EM, Wang A, Wilson AC (1982). Mitochondrial DNA sequences of primates: Tempo and mode of evolution.. J Mol Evol.

[29] Liu F, Miyamoto M, Freire N, Ong P, Tennant M (2001). Molecular and morphological supertrees for eutherian (placental) mammals.. Science.

[30] Raina SZ, Faith JJ, Disotell TR, Seligmann H, Stewart CB (2005). Evolution of base-substitution gradients in primate mitochondrial genomes.. Genome Res.

[31] Kullberg M, Nilsson M, Arnason U, Harley E, Janke A (2006). Housekeeping genes for phylogenetic analysis of Eutherian relationships.. Mol Biol Evol.

[32] Musto H, Cacciò S, Rodríguez-Maseda H, Bernardi G (1997). Compositional constraints in the extremely GC-poor genome of *Plasmodium falciparum*.. *Mem* Inst Oswaldo Cruz.

[33] Murtagh F, Chambers JM, Gordesch J, Klas A, Lebart L, Sint PP (1985). Multidimensional clustering algorithms.. COMPSTAT lectures vol 4.

[34] Kamimura T, Shimodaira H, Imoto S, Kim S, Tashiro K (2003). Multiscale bootstrap analysis of gene networks based on Bayesian networks and nonparametric regression.. Genome Inform.

[35] Kingsford C, Nagarajan, Salzberg S (2009). 2009 swine-origin influenza A (H1N1) resembles previous influenza isolates.. PLoS ONE.

