# Correction: A Novel Method of Characterizing Genetic Sequences: Genome Space with Biological Distance and Applications

**DOI:** 10.1371/annotation/22351496-73dc-4205-9d9a-95a821ae74ca

**Published:** 2011-03-09

**Authors:** Mo Deng, Chenglong Yu, Qian Liang, Rong L. He, Stephen S.-T. Yau

There is an update to the fifth author's affiliation. Stephen S.-T. Yau's affiliation is: "Department of mathematical sciences, Tsinghua University, Beijing, P.R. China"

