# Supplementary material for: A Novel Method of Characterizing Genetic Sequences: Genome Space with Biological Distance and Applications
Source: PLoS One. 2011 Mar 2;6(3):e17293. doi: 10.1371/journal.pone.0017293 (PMC3047556; doi:10.1371/journal.pone.0017293)
Supplement: Supporting Information S1 — contains the complete proof of the correspondence theorem, the bootstrapping analysis on A H1N1 genomes, distribution of the distance between each pair of random shuffled genomes under simulation, clustering of the segmented gene PB2, computational time chart of natural vector method, ClustalW2, MUSCLE and MAFFT, and the Genbank ID of the data used in this paper. (PDF) [file pone.0017293.s001.pdf]

A novel method of characterizing genetic sequences: genome space with biological distance and applications

Mo Deng, Chenglong Yu, Qian Liang, Rong L. He and Stephen Yau

Supporting Information S1

This supporting information has been provided by the authors to give readers additional information about their work.

1. The proof of the Theorem

Theorem: Suppose a DNA sequence has the number n of nucleotides. Then the correspondence between the DNA sequence and its natural vector

$$< n_A, \mu_A, n_C, \mu_C, n_G, \mu_G, n_T, \mu_T, D_2^A, \ldots, D_{n_A}^A, D_2^C, \ldots, D_{n_C}^C,$$

$$D_2^G, \ldots, D_{n_G}^G, D_2^T, \ldots, D_{n_T}^T >$$

is one-to-one, where $n = n_A + n_C + n_G + n_T$.

Proof. To prove the theorem, first we need prove that for any given proper DNA natural vector, we can recover the corresponding DNA sequence. Let us first denote $z_{[k]i} = s[k][i] - \mu_k$, then the normalized central moments can be simplified as:

$$D_j^k = \sum_{i=1}^{n_k} \frac{z_{[k]i}^j}{n_k^{j-1} n^{j-1}}, j = 1, 2, \ldots, n_k,$$

where n is the total length of the DNA sequence. If we can find the value of each z_i, the DNA sequence can be recovered. To solve for z_i, let $\delta_{[k]j} = D_j^k n_k^{j-1} n^{j-1}$, then the δ_j can be obtained by D_j^k and n_k (generally we assume each $n_k \geq 2$). For a given natural vector, n_k is known for each nucleic base A, C, G or T. So we need to solve for $z_{[k]i}$ corresponding to one of the n_k. Clearly $\delta_{[k]j}$ and $z_{[k]i}$ have the relation as below:

$$\begin{cases} \delta_{[k]1} = z_{[k]1} + z_{[k]2} + \ldots + z_{[k]n_k} \\ \delta_{[k]2} = z_{[k]1}^2 + z_{[k]2}^2 + \ldots + z_{[k]n_k}^2 \\ \ldots\ldots\ldots\ldots\ldots\ldots\ldots\ldots\ldots\ldots \\ \delta_{[k]n_k} = z_{[k]1}^{n_k} + z_{[k]2}^{n_k} + \ldots + z_{[k]n_k}^{n_k} \end{cases}$$

Assume that $z_{[k]1}, z_{[k]2}, \ldots, z_{[k]n_k}$ are roots of a symmetric polynomial. We choose nucleotide A as an illustration. Then $z_1, z_2, \ldots, z_{n_A}$ are roots of a symmetric polynomial $a_0 + a_1 z + a_2 z^2 + \ldots + a_{n_A} z^{n_A} = (z - z_1)(z - z_2) \ldots (z - z_{n_A})$. Let p_d $(d = 1, 2, \ldots, n)$ be the elementary symmetric polynomials in $z_1, z_2, \ldots, z_n$, i.e.,

$$p_1 = \sum_1^{n_A} z_i, p_2 = \sum_{i<j} z_i z_j, p_3 = \sum_{i<j<l} z_i z_j z_l,$$

$$\ldots, p_{n_A} = z_1 z_2 \ldots z_n$$

Then

$$p_1 = -a_{n_A-1}, p_2 = a_{n_A-2}, \ldots, p_{n_A} = (-1)^{n_A} a_0.$$

By using Newton's famous identities (Jacobson, 1974):

$$\delta_d - p_1\delta_{d-1} + \ldots + (-1)^{d-1}p_{d-1}\delta_1 + (-1)^d p_d = 0,$$

where $d = 1, 2, \ldots, n$, p_d is the elementary symmetric polynomials in $z_1, z_2, \ldots, z_{n_A}$. a_i can be obtained by δ_j as shown below:

$$\begin{cases} a_{n_A} &= 1 \\ a_{n_A-1} &= (-1)\delta_1 \\ a_{n_A-2} &= \frac{1}{2}(\delta_1^2 - \delta_2) \\ a_{n_A-3} &= (-1)^3\frac{1}{6}(\delta_1^3 - 3\delta_1\delta_2 + 2\delta_3) \\ a_{n_A-4} &= \frac{1}{24}(\delta_1^4 - 6\delta_1^2\delta_2 + 3\delta_2^2 + 8\delta_1\delta_3 - 6\delta_4) \\ \ldots\ldots\ldots\ldots\ldots\ldots\ldots\ldots\ldots\ldots\ldots\ldots\ldots \end{cases}$$

I.e., $a_{n_A} = 1, a_{n_A-1} = -p_1, a_{n_A-2} = p_2, \ldots.$ As a result, the coefficients of the symmetric polynomial $a_0 + a_1 z + a_2 z^2 + \ldots + z^{n_A} = (z - z_1)(z - z_2)\ldots(z - z_{n_A})$ can be confirmed, and then all roots can be obtained. Next we need to identify each root $z_1, z_2, \ldots, z_{n_A}$.

$z_i - z_{i+l} = s[A][i] - s[A][i+l]$. For any $l > 0$, $s[A][i] < s[A][i+l]$ by the definition of $s[A][i]$. It is obviously that $z_i < z_{i+l}$. As a consequence z_i is strictly increasing and each root can be identified by this property, which means each value of $s[A][i]$ can be obtained. Since μ_A is known for a given natural vector, $s[A][i]$ can also be obtained.

Similarly, we can find all $s[k][i]$ for $k = C, G, T$, respectively. Therefore, the unique corresponding DNA sequence can be recovered based on all $s[k][i]$, k is A, C, G, T.

On the other hand, given a DNA sequence, the number of each nucleic acid A, C, G and T and the distance of each nucleotide to the origin are fixed. Based on this information, we can compute T_k and μ_k. So it is easy to construct a natural vector. Clearly, any two different DNA sequences are distinct in length, number of each nucleic base or nucleotide arrangement. Thus, the two corresponding natural vectors are completely different.

Therefore, we have successfully proved that the correspondence between a DNA sequence and its distance vector is one-to-one.□

Remark We can see that $D_1^k = \sum_{i=1}^{n_k} z_i$, thus

$$D_1 = \sum_{i=1}^{n_k}(s[k][i] - \mu_k)$$

$$= T_k - n_k(\frac{T_k}{n_k}) = 0$$

So the natural vector sequence does not need D_1^k.

For assessing the uncertainty in hierarchical cluster analysis on influenza A (H1N1) genomes, p-values are calculated via multiscale bootstrap resampling for each cluster in hierarchical clustering. For a cluster with AU p-value> 0.95, the hypothesis that the cluster does not exist, is rejected with significance level 0.05; roughly speaking, we can think that these highlighted clusters do exist not only caused by sampling error, but can stably be observed if we increase the number of observation (Figure S1(a), S1(b) shown at the end of this Supporting Information S1).

2.1 Distribution of pair-wise distance L under simulation

It is an interesting problem to know the distribution of distance L of natural vectors for random sequences. This is performed by taking any sequence and generating a large number of shuffles of the sequence so that the total numbers of each nucleotide base are

preserved. For example, we select a human mitochondrial genome(GenBank ID: V00662), and then shuffle it 1000 times so that the total numbers of A, C, G, T are preserved. We first use 12 dimensional natural vectors. Therefore, 500500 different Ls will be generated, and then we plot the histogram of those Ls and estimate this probability distribution directly from the Ls by plotting a density curve. The histogram shows the distribution of frequency of those Ls. Then we take 16 dimensional natural vectors and draw the histogram again. There are no obvious differences between those two histograms, which means 12 dimensional natural vectors are stable. The histogram (Figure S2(a),S2(b)) was shown at the end of this Supporting Information S1.

2.2 Simulation of Gene Rearrangements

Gene rearrangements play important roles in evolution. The order of genes and transcription regions are changed during evolution by gene rearrangements such as DNA inversions and transpositions, which do not affect the gene content of the chromosomes. For example, inversions of large genomic fragments are often observed even between closely related species. The phylogenetic analysis based on gene order is challenging as it requires detailed complex gene order data in genomes and intensive computation. In order to test whether our method is stable for genomic rearrangement, we consider the following simulated experiment. We choose a human mitochondrial genome denoted by human, and then inverse its two genes ATPase 6 and Cytochrome oxidase to get a simulated genome, which is denoted by human-inv. We can treat this new simulated genome as the result of the inversion of genes from the original human mitochondrial genome. Next we randomly generate a genome sequence which has the same length and nucleotide content as the original human genome, which we denote by human-ran. Thus, we have 3 genomes of the same length and nucleotide content: human, human-inv and human-ran. In addition, we also choose another mitochondrial genome, chimpanzee as comparison since chimpanzee and human are so close evolutionarily. By using the 16-dimensional natural vector, we calculate the distance among these 4 genomes and get a distance matrix in S.Table 5. In S.Table 5, we found that the distance between human and human-inv is as small as 1.7 units. This means that the gene-inverted genome still has a very short distance to the original genome even if some gene rearrangement happens in this genome. As a result, the original genome and its gene rearrangement genome cannot be treated separately by using our method since the evolutionarily very close genome of human is chimpanzee which has the distance of 16.88 units to human. More importantly, this simulation demonstrates that our method can be applied to do clustering or phylogenetic analysis. If two genetic sequences are close in the distance, they should be close in the evolutionary tree. For example, the distance between real human genomes and the genome of chimpanzee is 16.88 units. Since the distance between the original human genome and all shuffled genomes ranges from 114.49 to 1009.00 with the mean value of 190.60 units (Figure S2(a) and S(b)), all those randomly shuffled sequences cannot be clustered together with the human.

Table S1: Distance matrix of simulation of gene rearrangement

	human	human-inv	human-ran
human-inv	1.717323		
human-ran	127.27331	128.14110	
chimpanzee	16.87587	17.047305	104.02812

In this table, we find that the distance between human and human-inv is as small as 1.717

units compared with others. This means that the new produced genome still has a very short distance from the original genome even if some gene rearrangement happens in a genome.

2.3 Construction of natural vectors of genomes

We have already obtained a good numerical characterization (natural vector) to represent a DNA sequence. Now we will use this tool to construct a natural vector for genomes. It is known that the structure of genomes is very complicated. It may be single-stranded or double-stranded, and in a linear, circular or segmented structure. Thus, we should consider the different structures when constructing the natural vector for genomes.

For the simplest genome structures, linear single-strand forms, we can treat them as linear DNA sequences. That is, every genome corresponds to a general DNA sequence. Thus, we can utilize our method to construct the natural vector for genomes. In order to use whole genome information to make comparative analysis among genomes, we can use the first N dimensional natural vector

$$< n_A, n_C, n_G, n_T, \mu_A, \mu_C, \mu_G, \mu_T, D_2^A, D_2^C, D_2^G, D_2^T, \ldots, D_S^A, D_S^C, D_S^G, D_S^T >$$

of the whole natural vector of a genome sequence to represent a genome where $S = \frac{N}{4} - 1$. Thus, using the Euclidean distance between each pair of vectors for comparison, we can perform phylogenetic and clustering analysis for genome sequences.

For the circular single-strand genomes, the construction of natural vector of a genome is more complicated because we do not know which point is the starting point in this circular DNA sequence. In this case, we treat every point as the starting point in this circular sequence of length n, and then we get n linear single-strand genomes. For every linear single-strand genome sequence, we can compute its $(n + 4)$-dimensional natural vector. Then we take average to get a normalized vector. For circular single-strand genomes, we use the first N dimensional natural vector (as shown above) of this normalized vector of each genome to do clustering and phylogenetic analysis. For the double-stranded genomes, we need to state that the natural vector of reverse complementary sequence is not the same as the original sequence. Generally, for the double-stranded genomes, we treat them as two single-stranded genomes. We use the above method (linear or circular) to get two $(n+4)$-dimensional natural vectors for these two single-stranded sequences, and then take average for them to get a general natural vector

$$< n_A, n_C, n_G, n_T, \mu_A, \mu_C, \mu_G, \mu_T, D_2^A, D_2^C, D_2^G, D_2^T, \ldots, D_{n_A}^A, D_{n_C}^C, D_{n_G}^G, D_{n_T}^T >$$

By using the first N dimensional natural vector of this general natural vector for a genome, we can do clustering analysis for those genomes. Here we need to point out that the two strands of some genomes (e.g., mitochondrial genomes, some bacterial genomes) are differentiated by their nucleotide content, which are called the heavy strand and the light strand respectively. The two strands have different masses because one has a higher proportion of heavier nucleic acids and its complement has a lower proportion. In this case, we just treat them as the single-stranded (by using the heavy strand) genomes to construct the natural vectors. For a genome consisting of k segments, we compute the natural vector for each DNA and then concatenate these k natural vectors to represent the natural vector for a segmented genome. For example, each segmented influenza A (H1N1) genome consists of 8 segments. So we can compute the natural vector for each segment and then concatenate these 8 natural vectors together. In our experiment, a 12-dimensional natural vector can characterize each A (H1N1) segment, then 96-dimensional natural vector can be used to characterize an influenza A (H1N1) genome.

Table S2: THE LIST OF 59 A H1N1 GENOMES.

No. on tree	Genome Strain Designation
1	Influenza A virus(A/California/04/2009(H1N1))
2	Influenza A virus(A/New York/18/2009(H1N1))
3	Influenza A virus(A/Canada-ON/RV1527/2009(H1N1))
4	Influenza A virus(A/Mexico/InDRE4487/2009(H1N1))
5	Influenza A virus(A/Texas/09/2009(H1N1))
6	Influenza A virus(A/California/14/2009(H1N1))
7	Influenza A Virus(A/New York/1669/2009(H1N1))
8	Influenza A Virus(A/New York/1682/2009(H1N1))
9	Influenza A virus(A/Canada-AB/RV1532/2009(H1N1))
10	Influenza A virus(A/Canada-NS/RV1536/2009(H1N1))
11	Influenza A virus(A/Canada-NS/RV1538/2009(H1N1))
12	Influenza A virus(A/Canada-ON/RV1526/2009(H1N1))
13	Influenza A virus(A/Canada-ON/RV1529/2009(H1N1))
14	Influenza A virus(A/Mexico/inDRE4114/2009(H1N1))
15	Influenza A virus(A/New York/3008/2009(H1N1))
16	Influenza A virus(A/New York/3014/2009(H1N1))
17	Influenza A virus(A/New York/3099/2009(H1N1))
18	Influenza A virus(A/swine/Alberta/OTH-33-8/2009(H1N1))
19	Influenza A virus(A/Hamburg/4/2009(H1N1))
20	Influenza A virus(A/England/195/2009(H1N1))
21	Influenza A virus (A/swine/Alberta/56626/03(H1N1))
22	Influenza A virus (A/swine/California/T9001707/1991(H1N1))
23	Influenza A virus (A/swine/OH/511445/2007(H1N1))
24	Influenza A virus (A/swine/Memphis/1/1990(H1N1))
25	Influenza A virus (A/swine/Iowa/31483/1988(H1N1))
26	Influenza A virus (A/swine/Ontario/55383/04(H1N2))
27	Influenza A virus (A/swine/Kansas/3228/1987(H1N1))
28	Influenza A virus (A/swine/Wisconsin/10/1998(H1N1)
29	Influenza A virus (A/swine/Denmark/WVL9/1993(H1N1))
30	Influenza A virus (A/swine/Spain/50047/2003(H1N1))
31	Influenza A virus (A/swine/England/WVL15/1997(H1N1))
32	Influenza A virus (A/swine/France/WVL4/1985(H1N1))
33	Influenza A virus (A/swine/Italy/671/1987(H1N1))
34	Influenza A virus (A/swine/Tianjin/01/2004(H1N1))
35	Influenza A virus (A/swine/Ratchaburi/NIAH1481/2000(H1N1))
36	Influenza A virus (A/swine/Nakhon pathom/NIAH586-1/2005(H3N2))
37	Influenza A virus (A/duck/NJ/7717-70/1995(H1N1))
38	Influenza A virus (A/blue winged teal/TX/27/2002(H1N1))
39	Influenza A virus (A/mallard/Maryland/42/2003(H1N1))
40	Influenza A virus (A/mallard/MN/330/1999(H3N1))
41	Influenza A virus (A/duck/Nanchang/4-165/2000(H4N6))
42	Influenza A virus (A/Duck/NY/185502/2002(H5N2))
43	Influenza A virus (A/chicken/Chis/15224/1997(H5N2))
44	Influenza A virus (A/duck/Italy/69238/2007(H1N1))
45	Influenza A virus (A/crested eagle/Belgium/01/2004(H5N1))

Table S2 cont

No. on tree	Genome Strain Designation
46	Influenza A virus (A/swan/Germany/R65/2006(H5N1))
47	Influenza A virus (A/Cygnus olor/Astrakhan/Ast05-2-1/2005(H5N1))
48	Influenza A virus (A/chicken/Crimea/08/2005(H5N1))
49	Influenza A virus (A/blue-winged teal/Ohio/1864/2006(H3N8))
50	Influenza A virus (A/chicken/Jiangsu/cz1/2002(H5N1))
51	Influenza A virus (A/egret/Hong Kong/757.2/2003(H5N1))
52	Influenza A virus (A/duck/Yokohama/aq10/2003(H5N1))
53	Influenza A virus (A/chicken/Korea/ES/03(H5N1))
54	Influenza A virus (A/Puerto Rico/8/34(H1N1))
55	Influenza A virus (A/New Caledonia/20/1999(H1N1))
56	Influenza A virus (A/Wisconsin/67/2005(H3N2))
57	Influenza A virus (A/New York/146/2000(H1N1))
58	Influenza A virus (A/Albany/20/1978(H1N1))
59	Influenza A virus (A/Wyoming/03/2003(H3N2))

Table S3: HRV Genomes

Number	Genome names on tree	Accession Number	species	seq length
1	cva-13	AF499637	HEV-C	7458
2	cva-21	AF546702	HEV-C	7406
3	pv-1m	V01149	HEV-C	7440
4	pv-2	M12197	HEV-C	7440
5	pv-3	K01392	HEV-C	7431
6	cb1	M16560	HEV-B	7389
7	cb2	AF081485	HEV-B	7403
8	cb3	M33854	HEV-B	7399
9	hrv-03	DQ473485	HRV-B	7208
10	hrv-04	DQ473490	HRV-B	7212
11	hrv-05	FJ445112	HRV-B	7212
12	hrv-06	DQ473486	HRV-B	7216
13	hrv-14	L05355	HRV-B	7212
14	hrv-17	EF173420	HRV-B	7219
15	hrv-26	FJ445124	HRV-B	7211
16	hrv-27	FJ445186	HRV-B	7217
17	hrv-35	FJ445187	HRV-B	7224
18	hrv-37	EF173423	HRV-B	7216
19	hrv-42	FJ445130	HRV-B	7223
20	hrv-48	DQ473488	HRV-B	7214
21	hrv-52	FJ445188	HRV-B	7216
22	hrv-69	FJ445151	HRV-B	7211
23	hrv-70	DQ473489	HRV-B	7223
24	hrv-72	FJ445153	HRV-B	7216
25	hrv-79	FJ445155	HRV-B	7224
26	hrv-83	FJ445161	HRV-B	7230
27	hrv-84	FJ445162	HRV-B	7201
28	hrv-86	FJ445164	HRV-B	7213
29	hrv-91	FJ445168	HRV-B	7221
30	hrv-92	FJ445169	HRV-B	7233

31	hrv-93	EF173425	HRV-B	7215
32	hrv-97	FJ445172	HRV-B	7207
33	hrv-99	FJ445174	HRV-B	7208
34	hrv-01	FJ445111	HRV-A	7137
35	hrv-02	X02316	HRV-A	7102
36	hrv-07	FJ445176	HRV-A	7146
37	hrv-08	FJ445113	HRV-A	7108
38	hrv-09	FJ445177	HRV-A	7132
39	hrv-10	FJ445178	HRV-A	7137
40	hrv-11	EF173414	HRV-A	7125
41	hrv-12	EF173415	HRV-A	7124
42	hrv-13	FJ445116	HRV-A	7140
43	hrv-15	DQ473493	HRV-A	7134
44	hrv-16	L24917	HRV-A	7124
45	hrv-18	FJ445118	HRV-A	7119
46	hrv-19	FJ445119	HRV-A	7135
47	hrv-20	FJ445120	HRV-A	7163
48	hrv-21	FJ445121	HRV-A	7134
49	hrv-22	FJ445122	HRV-A	7129
50	hrv-23	DQ473497	HRV-A	7025
51	hrv-24	FJ445190	HRV-A	7132
52	hrv-25	FJ445123	HRV-A	7126
53	hrv-28	DQ473508	HRV-A	7148
54	hrv-29	FJ445125	HRV-A	7123
55	hrv-30	FJ445179	HRV-A	7099
56	hrv-31	FJ445126	HRV-A	7131
57	hrv-32	FJ445127	HRV-A	7133
58	hrv-33	FJ445128	HRV-A	7133
59	hrv-34	FJ445189	HRV-A	7119
60	hrv-36	DQ473505	HRV-A	7141
61	hrv-38	FJ445180	HRV-A	7136
62	hrv-39	AY751783	HRV-A	7136
63	hrv-40	FJ445129	HRV-A	7138
64	hrv-41	DQ473491	HRV-A	7145
65	hrv-43	FJ445131	HRV-A	7129
66	hrv-44	DQ473499	HRV-A	7123
67	hrv-45	FJ445132	HRV-A	7114
68	hrv-46	DQ473506	HRV-A	7149
69	hrv-47	FJ445133	HRV-A	7132
70	hrv-49	DQ473496	HRV-A	7109
71	hrv-50	FJ445135	HRV-A	7118
72	hrv-51	FJ445136	HRV-A	7152
73	hrv-53	DQ473507	HRV-A	7143
74	hrv-54	FJ445138	HRV-A	7134
75	hrv-55	DQ473511	HRV-A	7036
76	hrv-56	FJ445140	HRV-A	7136
77	hrv-57	FJ445141	HRV-A	7134
78	hrv-58	FJ445142	HRV-A	7140

79	hrv-59	DQ473500	HRV-A	7135
80	hrv-60	FJ445143	HRV-A	7139
81	hrv-61	FJ445144	HRV-A	7139
82	hrv-62	FJ445145	HRV-A	7131
83	hrv-63	FJ445146	HRV-A	7141
84	hrv-64	FJ445181	HRV-A	7129
85	hrv-65	FJ445147	HRV-A	7162
86	hrv-66	FJ445148	HRV-A	7139
87	hrv-67	FJ445149	HRV-A	7135
88	hrv-68	FJ445150	HRV-A	7164
89	hrv-71	FJ445152	HRV-A	7161
90	hrv-73	DQ473492	HRV-A	7140
91	hrv-74	DQ473494	HRV-A	7120
92	hrv-75	DQ473510	HRV-A	7137
93	hrv-76	FJ445182	HRV-A	7128
94	hrv-77	FJ445154	HRV-A	7136
95	hrv-78	FJ445183	HRV-A	7145
96	hrv-80	FJ445156	HRV-A	7138
97	hrv-81	FJ445157	HRV-A	7116
98	hrv-82	FJ445160	HRV-A	7123
99	hrv-85	FJ445163	HRV-A	7140
100	hrv-88	DQ473504	HRV-A	7143
101	hrv-89	FJ445184	HRV-A	7152
102	hrv-90	FJ445167	HRV-A	7124
103	hrv-94	FJ445185	HRV-A	7132
104	hrv-95	FJ445170	HRV-A	7110
105	hrv-96	FJ445171	HRV-A	7134
106	hrv-98	FJ445173	HRV-A	7133
107	hrv-100	FJ445175	HRV-A	7140
108	qpm	EF186077	HRV-C	6917
109	nat001	EF077279	HRV-C	7079
110	c024	EF582385	HRV-C	7099
111	nat045	EF077280	HRV-C	7015
112	c026	EF582387	HRV-C	7086
113	c025	EF582386	HRV-C	7114

Table S4: THE LIST OF 21 GENOMES USED FOR COMPARISONS

A(H1N1)-1	Influenza A virus(A/New York/18/2009(H1N1))
A(H1N1)-2	Influenza A virus(A/Canada-ON/RV1527/2009(H1N1))
A(H1N1)-3	Influenza A virus(A/Mexico/InDRE4487/2009(H1N1))
A(H1N1)-4	Influenza A virus(A/Texas/09/2009(H1N1))
A(H1N1)-5	Influenza A virus(A/California/14/2009(H1N1))
A(H1N1)-6	Influenza A Virus(A/New York/1669/2009(H1N1))
swine1	Influenza A virus (A/swine/Alberta/56626/03(H1N1))
swine2	Influenza A virus (A/swine/California/T9001707/1991(H1N1))
swine3	Influenza A virus (A/swine/Nebraska/123/1977(H1N1))
swine4	Influenza A virus (A/swine/Memphis/1/1990(H1N1))
swine5	Influenza A virus (A/swine/Iowa/31483/1988(H1N1))

swine6	Influenza A virus (A/swine/Ontario/55383/04(H1N2))
seasonal1	Influenza A virus (A/Puerto Rico/8/34(H1N1))
seasonal2	Influenza A virus (A/New Caledonia/20/1999(H1N1))
seasonal3	Influenza A virus (A/Wisconsin/67/2005(H3N2))
avian1	Influenza A virus (A/duck/NJ/7717-70/1995(H1N1))
avian2	Influenza A virus (A/blue winged teal/TX/27/2002(H1N1))
avian3	Influenza A virus (A/mallard/Maryland/42/2003(H1N1))
avian4	Influenza A virus (A/mallard/MN/330/1999(H3N1))
avian5	Influenza A virus (A/blue-winged teal/Ohio/1864/2006(H3N8))
avian6	Influenza A virus (A/Duck/NY/185502/2002(H5N2))

Table S5: GenBank accession numbers of gene PB2 used in genetic analyses.

No. on tree	GenBank ID.	Strain designation
1	M73515	A/swine/Iowa/15/1930(H1N1)
2	M55469	A/swine/1976/1931(H1N1)
3	CY026146	A/Wisconsin/301/1976(H1N1)
4	DQ280205	A/swine/Ontario/55383/04(H1N2)
5	DQ280189	A/swine/Ontario/57561/03(H1N1)
6	CY033629	A/New Caledonia/20/1999(H1N1)
7	CY034123	A/Wisconsin/67/2005(H3N2)
8	EU026109	A/duck/NY/13152-13/1994(H1N1)
9	AY619970	A/swine/Ontario/42729A/01(H3N3)
10	EU026021	A/mallard/MD/161/2002(H1N1)
11	AY619954	A/swine/Saskatchewan/18789/02(H1N1)
12	EU880827	A/turkey/CA/358533/2005(H4N8)
13	AF285892	A/Swine/Ontario/01911-1/99 (H4N6)
14	AF455736	A/Swine/Indiana/P12439/00 (H1N2)
15	AF250131	A/Swine/Indiana/9K035/99 (H1N2)
16	EU301177	A/swine/Korea/JNS06/2004(H3N2)
17	EU015993	A/swine/Guangxi/13/2006(H1N2)
18	AF455733	A/Swine/North Carolina/93523/01 (H1N2)
19	AY233387	A/duck/NC/91347/01(H1N2)
20	GQ160597	A/Nebraska/03/2009(H1N1)
21	GQ168878	A/New York/15/2009(H1N1)
22	GQ168876	A/New York/10/2009(H1N1)
23	FJ984351	A/New York/18/2009(H1N1)
24	GQ117021	A/New York/22/2009(H1N1)
25	GQ117035	A/California/14/2009(H1N1)
26	GQ149632	A/Mexico/4604/2009(H1N1)
27	GQ168870	A/Indiana/09/2009(H1N1)
28	GQ117076	A/Arizona/02/2009(H1N1)
29	FJ998206	A/Mexico/InDRE4487/2009(H1N1)
30	CY039908	A/New York/1682/2009(H1N1)
31	GQ132138	A/Mexico/InDRE4114/2009(H1N1)
32	GQ162180	A/Mexico/4108/2009(H1N1)
33	GQ149617	A/Mexico/4486/2009(H1N1)
34	GQ162168	A/Mexico/4603/2009(H1N1)
35	FJ966079	A/California/04/2009(H1N1)

36	FJ966976	A/California/07/2009(H1N1)
37	FJ984365	A/California/08/2009(H1N1)
38	GQ117089	A/Texas/07/2009(H1N1)
39	GQ117070	A/Minnesota/02/2009(H1N1)
40	FJ966955	A/California/05/2009(H1N1)
41	FJ966963	A/California/06/2009(H1N1)
42	GQ117047	A/Texas/08/2009(H1N1)
43	GQ168885	A/Texas/04/2009(H1N1)
44	GQ168879	A/Texas/06/2009(H1N1)
45	GQ117027	A/Texas/09/2009(H1N1)
46	AF342824	A/Wisconsin/10/98(H1N1)
47	EU798929	A/swine/Korea/CAS05/2004(H3N2)
48	AF455737	A/Swine/Illinois/100085A/01 (H1N2)
49	DQ469987	A/swine/Ontario/33853/2005(H3N2)
50	DQ469971	A/swine/British Columbia/28103/2005(H3N2)
51	EU604691	A/swine/OH/511445/2007(H1N1)
52	DQ280213	A/swine/Ontario/53518/03(H1N1)
53	AB473548	A/duck/Mongolia/47/2001(H7N1)
54	AY676023	A/chicken/Korea/ES/03(H5N1)
55	DQ464357	A/swan/Germany/R65/2006(H5N1)
56	CY037965	A/swine/Belgium/WVL2/1983(H1N1)
57	CY038004	A/swine/England/WVL7/1992(H1N1)
58	EF101747	A/Philippines/344/2004(H1N2)
59	FJ415615	A/swine/Zhejiang/1/2007(H1N1)
60	CY009379	A/swine/Spain/33601/2001(H3N2)
61	CY010587	A/swine/Spain/53207/2004(H1N1)
62	CY009899	A/Swine/Spain/50047/2003(H1N1)
63	CY038020	A/swine/Denmark/WVL9/1993(H1N1)
64	AJ293920	A/Hong Kong/1774/99(H3N2)
65	AB434293	A/swine/Ratchaburi/NIAH550/2003(H1N1)
66	AB434285	A/swine/Ratchaburi/NIAH1481/2000(H1N1)
67	EF101754	A/Thailand/271/2005(H1N1)
68	AB434325	A/swine/Chachoengsao/NIAH587/2005(H1N1)

The clustering results of other 7 individual genes: PB1, PA, hemagglutinin HA, neuraminidase NA, nucleocapsid NP, matrix protein MP and nonstructural gene NS can be available upon request from authors.

Supporting Information Figure Legends

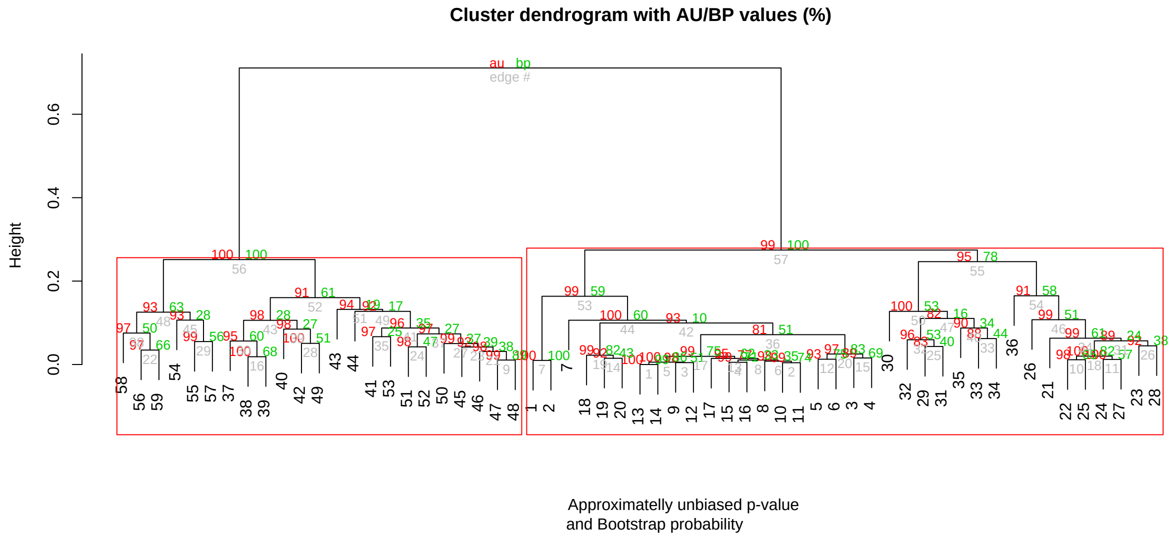

Figure S1(a): Genome analysis. Natural vector method and hierarchical clustering method are used to reconstruct the phylogenetic tree for nucleotide sequences of the whole genome sequences of selected influenza viruses. The robustness of individual nodes of the tree is assessed using a bootstrap resampling analysis with 1000 replicates. From the clustering result, we can see that the new swine influenza A (H1N1) viruses resemble the triple-reassortant swine influenza A virus and Eurasian classical swine lineages. For each cluster in the hierarchical clustering, p-values are calculated via multiscale bootstrap resampling. Red values are AU (approximately unbiased) p-values, and green values are BP (bootstrap probability) values. Clusters with AU larger than 95% are strongly supported by data. The hypothesis that "the cluster does not exist" is rejected with significance level 0.05; we can believe that these highlighted clusters exist and can be stably observed if we increase the number of observation. We conduct hierarchical cluster analysis with multiscale bootstrap with number of bootstrapping 1000 using average method and Euclidean distance. The new influenza A (H1N1) viruses resemble the swine virus (in the same cluster).

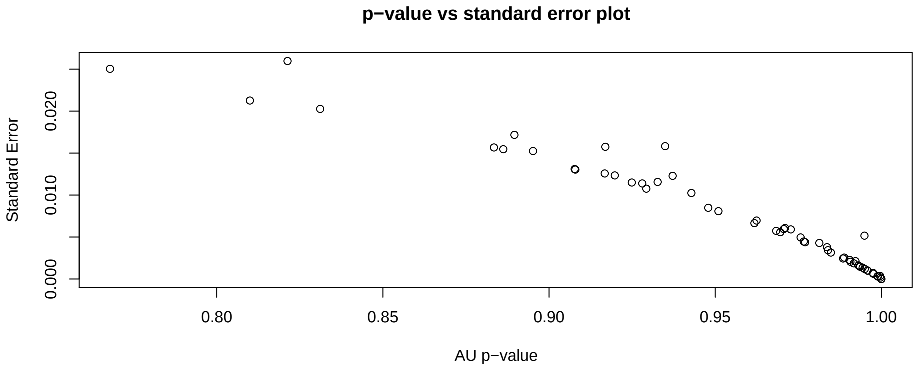

Figure S1(b): Std-error: the AU p-values themselves include sampling error, since they are also computed by a limited number of bootstrap samples (see figure 1 in main text of the paper). The standard errors of AU p-values were shown here. All clusters with AU p-values$\geq$ 95%, we can say that these highlighted clusters may stably be observed if we increase the number of observations (genomes). As the plot shows, all clusters have standard errors smaller than 0.05.

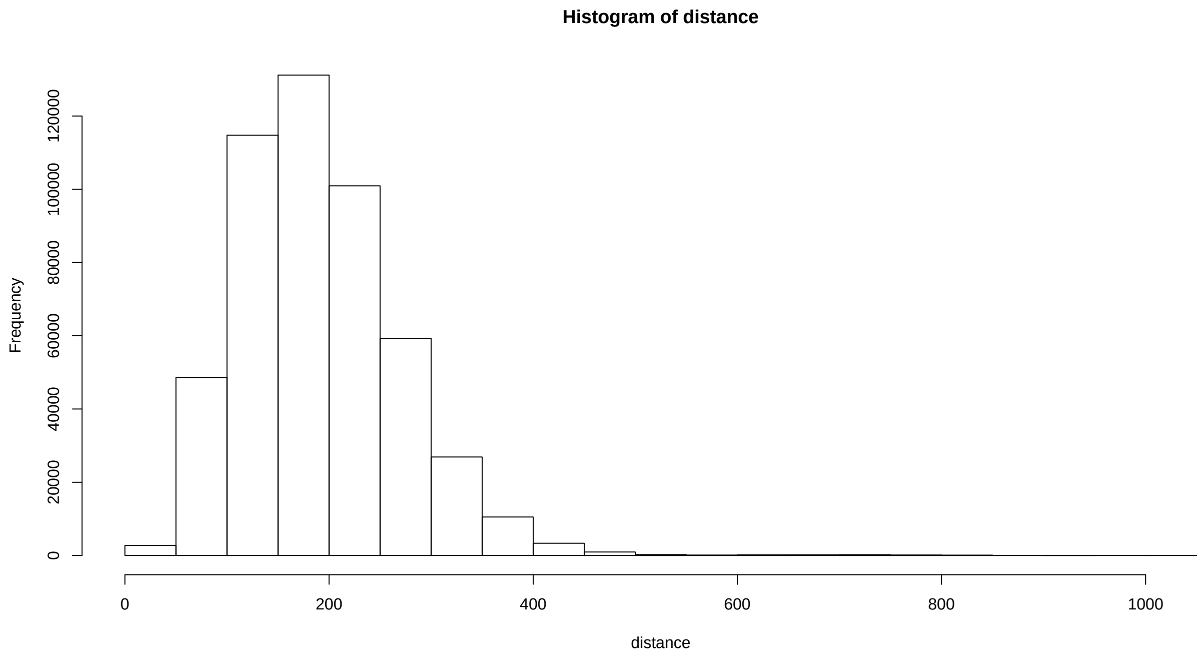

Figure S2(a): Distribution of frequency of pair-wise distance Ls under simulation.

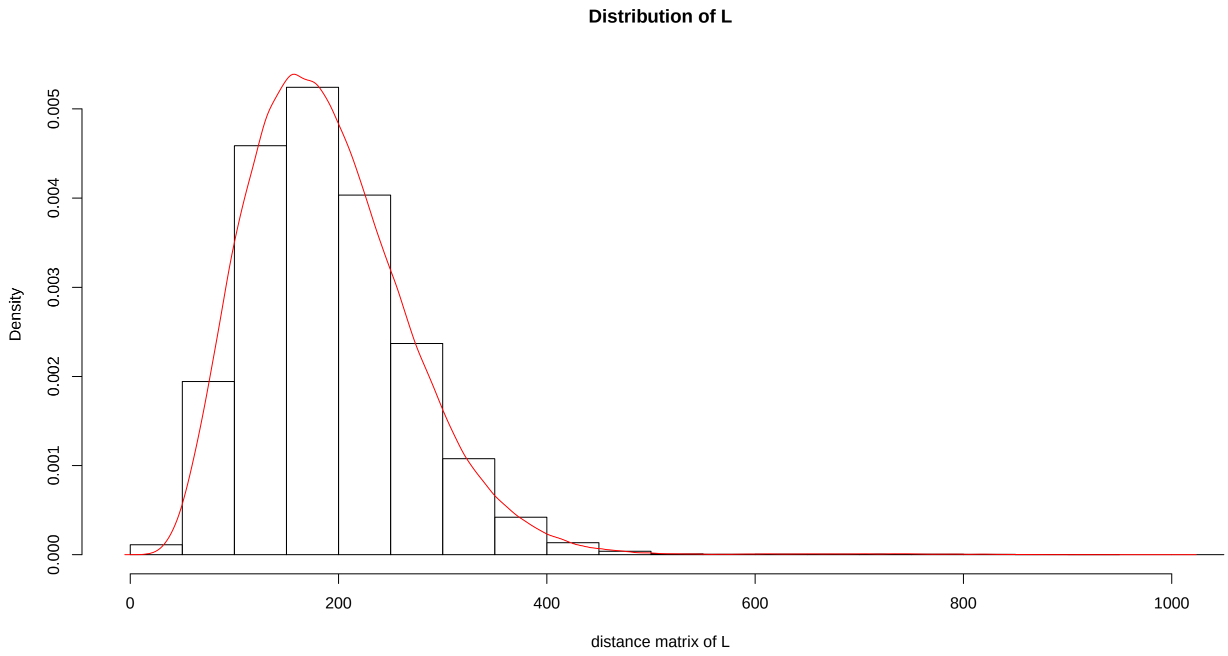

Figure S2(b): Density curve of pair-wise distance Ls under simulation.

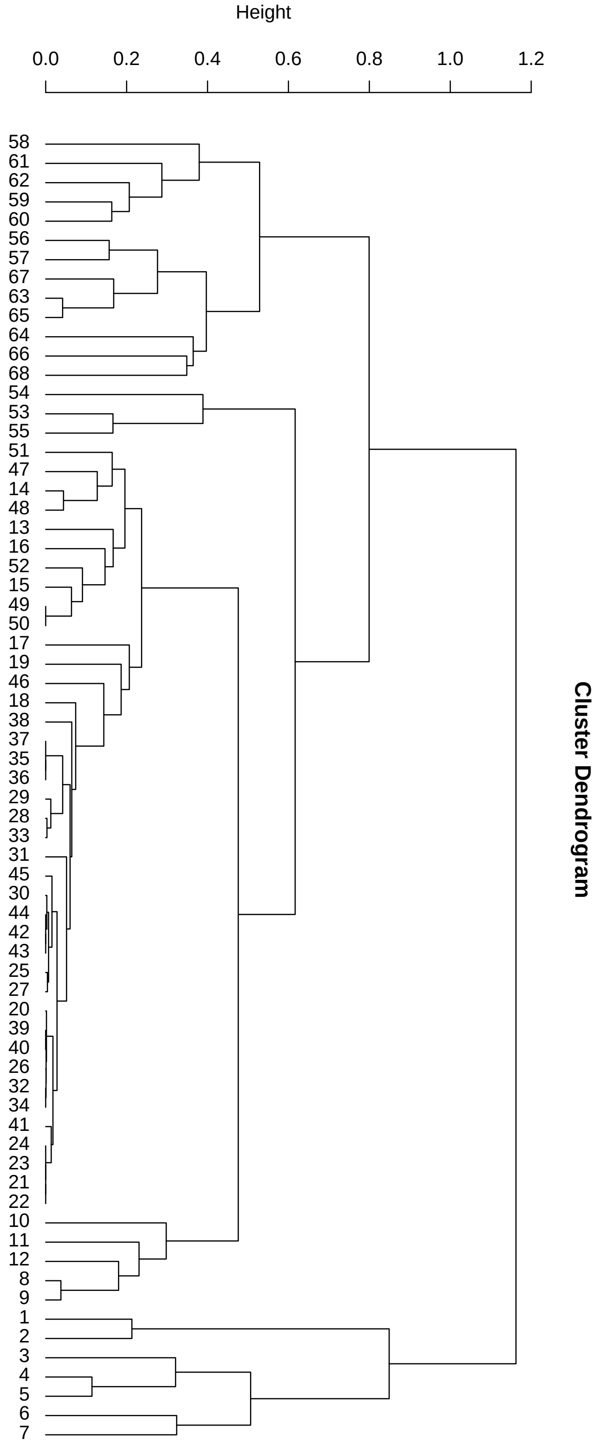

Figure S3: The clustering result by using natural vector method on PB2 complete coding segments of selected influenza viruses was shown here. The selected viruses are chosen to be representative from among all available relevant sequences in GenBank. Classical swine: 1-5; human seasonal (H1N1): 6-7; American avian: 8-12; triple reassortant swine: 13-19, 46-52; new swine influenza A (H1N1): 20-45; Eurasian avian: 53-55; Eurasian swine: 56-68. The result confirms that PB2 genes are similar to ones previously found in triple-reassortant swine influenza viruses circulating in pigs in North America. The gene information is provided in Table S5.

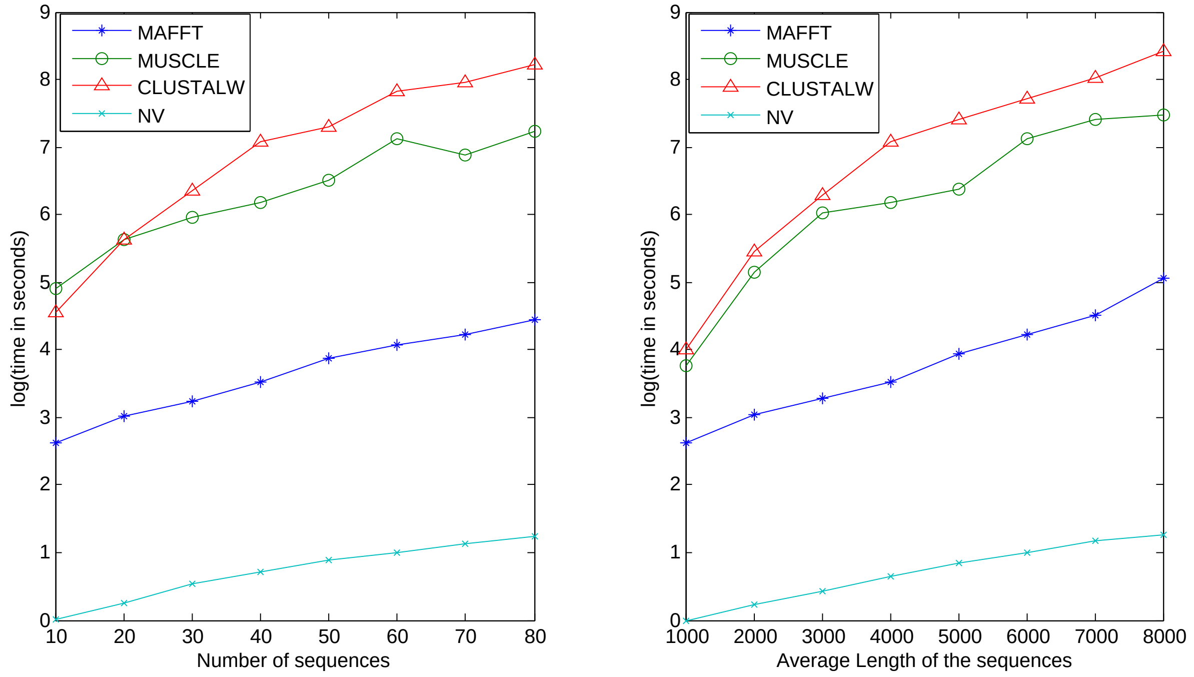

Figure S4: the first set includes 8 datasets (left sub-figure). Each dataset contains 10, 20, 30, 40, 50, 60, 70 and 80 sequences respectively, where the lengths of all the sequences are around 4000 bp. Another set is constructed with 8 datasets. Each single dataset has 40 sequences. The lengths of all sequences in these 8 datasets are 1000, 2000, 3000, 4000, 5000, 6000, 7000 and 8000 bp respectively (right sub-figure). We build the trees on each dataset of these two sets by using the four methods and record the time each method takes. The time of natural vector method increases linearly as the number of sequences or the length of sequences increase, whereas the acceleration of computation time of other three methods is much faster (y-axis is the logarithm of computation time).

Supporting Information S1's references

[1] Efron B., Tibshirani J.R. 1993. An introduction to the bootstrap.*Chapman&Hall, New York*. 456pp.

[2] Hogg R., et al. 2005. Introduction to Mathematical Statistics, 6th edition.*Pearson Prentice Hall*, 692pp.

[3] Jacobson N. 1974. Basic Algebra I. *W.H.Freeman, San Francisco*, 135pp.
